# Clinical Characteristics of Multisystem Inflammatory Syndrome in Adults: A Systematic Review

**DOI:** 10.1001/jamanetworkopen.2021.26456

**Published:** 2021-09-22

**Authors:** Pragna Patel, Jennifer DeCuir, Joseph Abrams, Angela P. Campbell, Shana Godfred-Cato, Ermias D. Belay

**Affiliations:** 1 CDC COVID-19 Response, Division of Emergency Operations, Center for Preparedness and Response, Centers for Disease Control and Prevention, Atlanta, Georgia; 2 Epidemic Intelligence Service, Centers for Disease Control and Prevention, Atlanta, Georgia

## Abstract

**Importance:**

Multisystem inflammatory syndrome in adults (MIS-A) has not been well described. Improved diagnosis and treatment of MIS-A might mitigate COVID-19 morbidity and mortality.

**Objective:**

To summarize the descriptive epidemiology and clinical characteristics of MIS-A.

**Evidence Review:**

This systematic review identified patients with MIS-A using 3 strategies: (1) literature review from May 1, 2020, to May 25, 2021, by searching MEDLINE, Embase, Global Health, CAB Abstracts, PsycINFO, CINAHL (Cumulative Index to Nursing and Allied Health Literature), Academic Search Complete, Scopus, World Health Organization Global COVID-19 Literature Database, and Google Scholar; (2) voluntary reports of MIS-A to the Centers for Disease Control and Prevention (CDC); and (3) reports among persons aged 18 to 20 years in the CDC surveillance system for MIS in children.

**Findings:**

Of 221 patients with MIS-A, the median age was 21 (interquartile range [IQR], 19-34) years, and 154 of 219 (70%) with data available were men. Sixty of 169 patients (36%) were non-Hispanic Black individuals, and 122 of 209 (58%) had no underlying comorbidity. One hundred two of 149 patients (68%) noted a previous symptomatic COVID-19–like illness (median, 28 [IQR, 20-36] days previously). Most patients with MIS-A presented with fever (197 of 205 [96%]), hypotension (133 of 220 [60%]), cardiac dysfunction (114 of 210 [54%]), shortness of breath (102 of 198 [52%]), and/or diarrhea (102 of 197 [52%]). The median number of organ systems involved was 5 (IQR, 4-6). Median hospital stay was 8 (IQR, 5-12) days; 115 of 201 patients (57%) were admitted to the intensive care unit; 101 of 213 (47%) required respiratory support, and 15 of 220 (7%) died. Most patients (176 of 195 [90%]) had elevated markers of coagulopathy and/or inflammation and a positive SARS-CoV-2 serologic finding (139 of 194 [72%]). Ten patients with MIS-A presented with Kawasaki disease.

**Conclusions and Relevance:**

These findings suggest that MIS-A is a serious hyperinflammatory condition that presents approximately 4 weeks after onset of acute COVID-19 with extrapulmonary multiorgan dysfunction.

## Key Points

**Question:**

What are the clinical characteristics of multisystem inflammatory syndrome in adults (MIS-A)?

**Findings:**

This systematic review of patients with MIS-A reported in the literature and to the US Centers for Disease Control and Prevention identified 221 patients worldwide. The syndrome presented approximately 4 weeks after acute COVID-19 with hyperinflammation and extrapulmonary multiorgan involvement that may be difficult to discern from acute biphasic COVID-19 and postacute sequelae of SARS-CoV-2 infection.

**Meaning:**

These findings suggest that MIS-A occurs in the postacute COVID-19 period with a heterogeneous clinical presentation likely owing to a dysregulated immune response.

## Teaser

This systematic review provides a comprehensive overview of the clinical characteristics of multisystem inflammatory syndrome in adults.

## Introduction

As SARS-CoV-2 infections persist in the US^1^ and many countries worldwide, it is important for clinicians and public health officials to learn from the early days of the pandemic to reduce morbidity and mortality. This includes recognizing manifestations of COVID-19 with serious sequelae that may be poorly understood, underreported, and, worrisomely, not diagnosed in a timely manner, such as various clinical manifestations of hyperinflammation among persons with SARS-CoV-2 infection.

After multisystem inflammatory syndrome in children (MIS-C) was first identified in April 2020, many physicians noted a similar syndrome occurring in adults.^2,3^ Recognition of MIS in adults (MIS-A) is complicated by the occurrence of other types of COVID-19–related hyperinflammation, which make MIS-A hard to distinguish from biphasic acute COVID-19 and postacute sequelae of SARS-CoV-2 infection. The temporal association of MIS-A with SARS-CoV-2 infection and antecedent acute COVID-19 is also unknown. Better characterization of MIS-A is important because the clinical manifestations, illness progression, and treatment may be distinct from those of other types of severe COVID-19, with and without hyperinflammation. We conducted a literature review and examined cases reported to the Centers for Disease Control and Prevention (CDC) to describe the clinical characteristics of MIS-A, including laboratory results and empirical treatments.

## Methods

To provide a comprehensive overview of MIS-A, we identified cases of MIS-A using 3 strategies: (1) a literature review of case reports; (2) examination of cases that clinicians and health departments in the US voluntarily reported to the CDC using the MIS-C case report form; and (3) assessment of cases of MIS-C among persons aged 18 to 20 years reported to the CDC through the MIS-C surveillance system, which captures MIS cases in the US among persons younger than 21 years using the MIS-C case definition.^4^ The MIS-C case definition includes the following: (1) an individual younger than 21 presenting with fever; (2) laboratory evidence of inflammation; (3) evidence of clinically severe illness requiring hospitalization with multisystem (>2) organ involvement (cardiac, renal, respiratory, hematologic, gastrointestinal, dermatologic, or neurological); (4) no plausible alternate diagnoses; and (5) positive results for current or recent SARS-CoV-2 infection by RT-PCR, serologic analysis, or antigen test or exposure to a suspected or confirmed COVID-19 case within the 4 weeks prior to the onset of symptoms. The MIS-C definition, except for the age criterion, was applied to identify patients with MIS-A from all sources, with at least 4 criteria needed for inclusion. The case report form includes information on patient demographics, including date of birth, underlying medical conditions, clinical findings, complications, laboratory test results, imaging findings, treatments, and outcomes (eAppendix in the Supplement). We included cases among persons aged 18 to 20 years from the MIS-C surveillance system in this analysis because our definition of adults was persons 18 years or older.^3^

The CDC librarian conducted our literature search for reported cases of MIS-A from May 1, 2020, to May 25, 2021, by searching the following databases: MEDLINE, Embase, Global Health, CAB Abstracts, PsycINFO, CINAHL (Cumulative Index to Nursing and Allied Health Literature), Academic Search Complete, Scopus, World Health Organization Global COVID-19 Literature Database, and Google Scholar. Search terms included *severe inflammation*, *multisystem*, *Kawasaki/Kawasaki-like*, *shock/hypotension*, *organ dysfunction*, *multisystem inflammatory syndrome*, *MIS-A*, and *adults* (eTable 1 in the Supplement). This activity was reviewed by the CDC and was conducted in a manner consistent with applicable federal law and CDC policy (eg, 45 CFR part 46.102[l][2]; 21 CFR part 56; 42 USC §241[d]; 5 USC §552a; and 44 USC §3501 et seq). The activity was determined to meet the requirements of public health surveillance as defined in 45 CFR 46.102(l)(2).

**Figure 1.**
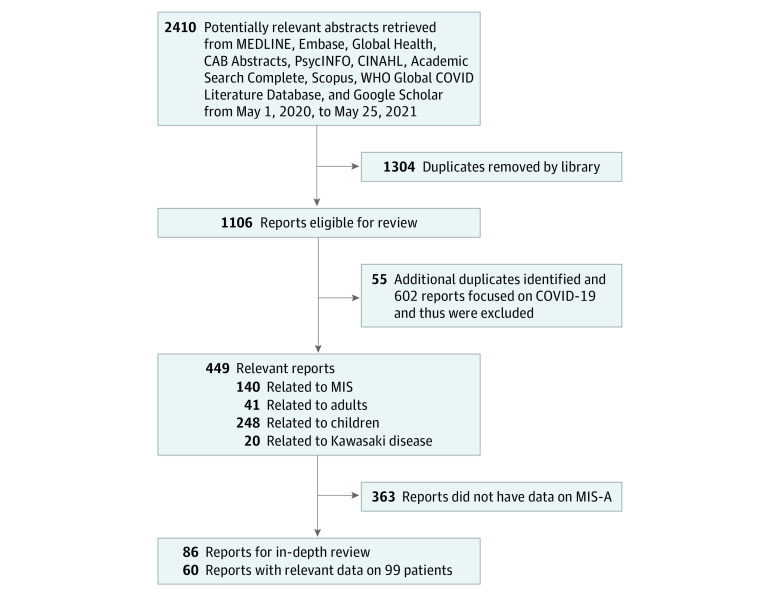
Selection of Studies Regarding Multisystem Inflammatory Syndrome in Adults (MIS-A) WHO indicates World Health Organization.

Of 2410 publications identified, all abstracts were screened using EndNote 20 (Clarivate) by 1 reviewer (P.P.) to identify cases of MIS-A based on the following inclusion criteria: patients 18 years or older who met at least 4 criteria of the MIS-C case definition, and the case was reported in English.^4^ Case reports were excluded for insufficient data, if the report was not in English, and if the report could not be accessed. If an abstract was not available, the full report was reviewed. Two clinician reviewers (P.P. and J.D.) then examined the selected articles for pertinent data and their references for additional relevant reports. Authors were contacted for clarification if needed. In total, 449 relevant articles were identified, and 86 were selected for in-depth review; of these, 60 had data for patients with MIS-A.^2,5,6,7,8,9,10,11,12,13,14,15,16,17,18,19,20,21,22,23,24,25,26,27,28,29,30,31,32,33,34,35,36,37,38,39,40,41,42,43,44,45,46,47,48,49,50,51,52,53,54,55,56,57,58,59,60,61,62,63^ Figure 1 summarizes the study selection process. We excluded 8 articles: 3 were not in English,^64,65,66^ 3 could not be located,^67,68,69^ 1 abstract did not have enough data for inclusion,^70^ and 1 reported case was deemed a misdiagnosis by 2 clinicians (P.P. and J.D.).^71^ Ten articles were case series and given a quality score of 4; all others were case reports and scored as 5 using the Quality Rating Scheme for Studies modified from the Oxford Centre for Evidence-Based Medicine for ratings of individual studies (eTable 2 in the Supplement).

Descriptive statistics on demographic, clinical, and laboratory features as well as previous illness and an estimate of the time from the initial SARS-CoV-2 infection to onset of MIS-A were summarized. Race and ethnicity data were self-reported and included to further discern COVID-19–associated health inequities. Previous COVID-19 was defined as an illness that was at least 7 days before the MIS-A presentation. Kawasaki disease was defined using the CDC case definition: illness in a patient with fever of 5 or more days’ duration (or fever until the date of administration of intravenous immunoglobulin if it is given before the fifth day of fever), and the presence of at least 4 of the following 5 clinical signs: rash, cervical lymphadenopathy (≥1.5 cm in diameter), bilateral conjunctival injection, oral mucosal changes, and peripheral extremity changes.^72^

We compared clinical characteristics of patients with MIS-A reported to the CDC with those of patients with MIS-C who were younger than 18 years in the MIS-C surveillance system using the χ^2^ test. We used Excel, version 2102 (Microsoft Corp), and SAS, version 9.4 (SAS Institute, Inc), for all analyses. Two-sided *P* < .05 indicated statistical significance.

## Results

A total of 221 patients with MIS-A were identified: 102 were from the CDC’s MIS-C surveillance system, of whom 55 were described in a recent publication^3^; 20 were from voluntary reports to the CDC, of whom 9 were included in a recent publication^2^; and 99 were from case reports published in the literature.^2,5,6,7,8,9,10,11,12,13,14,15,16,17,18,19,20,21,22,23,24,25,26,27,28,29,30,31,32,33,34,35,36,37,38,39,40,41,42,43,44,45,46,47,48,49,50,51,52,53,54,55,56,57,58,59,60,61,62,63^ Two clinician reviewers (P.P. and J.D.) examined cases in the literature to ensure there was no duplication with cases voluntarily reported to the CDC based on age, sex, and author of the publication. In addition to the US cases, we found 52 reported cases of MIS-A from 16 other countries, including the UK, Italy, Israel, India, Canada, Japan, Pakistan, the United Arab Emirates, Croatia, Norway, France, Spain, Belgium, Portugal, South Africa, and Uruguay.

Of the 221 patients with MIS-A, median age was 21 (interquartile range [IQR], 19-34) years; of whom, 99 patients drawn from the literature had a median age of 33 (IQR, 24-45) years. Among those with data available, 154 of 219 patients (70%) were men and 65 of 219 (30%) were women; 60 of 169 (36%) were non-Hispanic Black individuals; and 122 of 209 (58%) had no underlying comorbidity (Table). Of 149 patients, 102 (68%) noted a previous symptomatic COVID-19–like illness and recovered before presenting with MIS-A; other patients were assumed to have had asymptomatic acute SARS-CoV-2 infection. The median time from onset of symptoms of prior COVID-19–like illness to MIS-A was 28 (IQR, 20-36) days, with the longest reported period being 68 days in a 67-year-old man with cirrhosis and hypertension.

**Table.** Demographic, Clinical, and Laboratory Characteristics of 221 Patients With MIS-A

Characteristic	All patients (N = 221)		Reported to CDC (n = 122)		From the literature (n = 99)	
	Data, No. (%)	Records with complete data, No.	Data, No. (%)	Records with complete data, No.	Data, No. (%)	Records with complete data, No.
Demographic						
Male	154 (70)	219	85 (70)	122	69 (71)	97
Female	65 (30)	219	37 (30)	122	28 (29)	97
Age, median (IQR), y	21 (19-34)	221	19 (19-21)	122	33 (24-45)	99
Race and ethnicity						
Asian	12 (7)	169	6 (5)	114	6 (11)	55
Hispanic	50 (30)	169	36 (32)	114	14 (25)	55
Non-Hispanic Black	60 (36)	169	38 (33)	114	22 (40)	55
Non-Hispanic White	41 (24)	169	30 (26)	114	11 (20)	55
Other^a^	6 (4)	169	4 (4)	114	2 (4)	55
History of SARS-CoV-2 infection						
Previous symptomatic COVID-19–like illness	102 (68)	149	59 (66)	90	43 (73)	59
Previous SARS-CoV-2 infection by symptoms and/or any testing results	139 (79)	175	59 (66)	90	80 (94)	85
Time since onset of previous symptomatic COVID-19–like illness, median (IQR), d	28 (20-36)	100	26 (13-35)	59	29 (23-42)	41
Clinical characteristics						
Presence of fever^b^	197 (96)	205	119 (98)	122	78 (94)	83
Underlying medical conditions	87 (42)	209	51 (42)	122	36 (41)	87
Obesity	54 (26)	209	37 (30)	122	17 (20)	87
Chronic lung disease	17 (8)	209	13 (11)	122	4 (5)	87
No. of organ systems involved						
Median (IQR)	5 (4-6)	221	5 (5-6)	122	5 (4-6)	99
2-3	46 (21)	221	14 (11)	122	32 (32)	99
4-5	116 (52)	221	66 (54)	122	50 (51)	99
≥6	55 (25)	221	39 (32)	122	16 (16)	99
Organ system involvement						
Gastrointestinal tract	182 (83)	218	111 (91)	122	71 (74)	96
Abdominal pain	95 (48)	199	68 (56)	122	27 (35)	77
Vomiting	86 (44)	197	68 (56)	122	18 (24)	75
Diarrhea	102 (52)	197	65 (53)	122	37 (49)	75
Cardiovascular	193 (87)	221	105 (86)	122	88 (89)	99
Chest pain, pressure, and/or tightness	59 (29)	201	45 (37)	122	14 (18)	79
Shock	114 (52)	218	55 (45)	122	59 (61)	96
Hypotension	133 (60)	220	65 (53)	122	68 (69)	98
Arrhythmia	36 (18)	205	26 (21)	122	10 (12)	83
Cardiac dysfunction	114 (54)	210	46 (38)	122	68 (77)	88
Myocarditis	61 (30)	205	33 (27)	122	28 (34)	83
Coronary artery dilatation or aneurysm	16 (8)	192	12 (10)	122	4 (6)	70
Pericarditis	6 (3)	199	4 (3)	122	2 (3)	77
Pericardial effusion	44 (25)	175	27 (22)	122	17 (32)	53
Mitral regurgitation	25 (14)	175	18 (15)	122	7 (13)	53
Dermatologic/mucocutaneous	100 (46)	218	50 (41)	122	50 (52)	96
Rash	83 (38)	217	43 (35)	122	40 (42)	95
Mucocutaneous lesions	35 (16)	216	14 (11)	122	21 (22)	94
Conjunctival injection	57 (26)	217	26 (21)	122	31 (33)	95
Hematologic	184 (92)	200	112 (92)	122	72 (92)	78
Arterial or venous thrombosis	9 (5)	195	7 (6)	122	2 (3)	73
Respiratory	159 (74)	215	96 (79)	122	63 (68)	93
Cough	74 (37)	200	58 (48)	122	16 (21)	78
Shortness of breath	102 (52)	198	64 (52)	122	38 (50)	76
Pneumonia	74 (37)	200	41 (34)	122	33 (42)	78
Acute respiratory distress syndrome	38 (20)	191	34 (28)	122	4 (6)	69
Pleural effusion	44 (23)	192	32 (26)	122	12 (17)	70
Neurological	103 (47)	218	71 (58)	122	32 (33)	96
Headache	84 (42)	202	65 (53)	122	19 (24)	80
Renal	79 (43)	185	45 (37)	122	34 (54)	63
Acute kidney injury	67 (39)	174	44 (36)	122	23 (44)	52
Other						
Periorbital edema	7 (4)	187	6 (5)	122	1 (2)	65
Cervical lymphadenopathy	29 (16)	187	9 (7)	122	20 (31)	65
SARS-CoV-2 testing						
Any positive laboratory test result	207 (98)	211	121 (100)	121	86 (96)	90
RT-PCR positive/serologic negative result^c^	49 (25)	194	41 (34)	121	8 (11)	73
RT-PCR negative/serologic positive result	77 (40)	194	33 (27)	121	44 (60)	73
RT-PCR positive/serologic positive result	62 (32)	192	47 (39)	121	15 (21)	71
Treatment						
Intravenous immunoglobulin	112 (55)	205	77 (63)	122	35 (42)	83
Corticosteroids	152 (74)	205	96 (79)	122	56 (67)	83
Antiplatelet medication	74 (38)	193	61 (50)	122	13 (18)	71
Anticoagulation medication	110 (57)	193	88 (72)	122	22 (31)	71
Vasoactive medications	110 (51)	214	59 (48)	122	51 (55)	92
Respiratory support, any	101 (47)	213	63 (52)	122	38 (42)	91
Intubation/mechanical ventilation	53 (25)	213	27 (22)	122	26 (29)	91
Immune modulators^d^	42 (21)	203	32 (26)	122	10 (12)	81
Convalescent plasma	4 (4)	92	1 (9)	11	3 (4)	81
Dialysis	15 (8)	193	8 (7)	122	7 (10)	71
Laboratory test result^e^						
Elevated fibrinogen level	93 (91)	102	70 (91)	77	23 (92)	25
Elevated D-dimer level	138 (91)	151	77 (87)	89	61 (98)	62
Elevated troponin level	127 (78)	163	62 (70)	88	65 (87)	75
Elevated BNP level	56 (74)	76	34 (79)	43	22 (67)	33
Elevated NT-proBNP level	53 (90)	59	30 (83)	36	23 (100)	23
Elevated C-reactive protein level	176 (90)	195	87 (83)	105	89 (99)	90
Elevated ferritin level	150 (91)	165	81 (87)	93	69 (96)	72
Elevated interleukin 6 level	61 (98)	62	34 (100)	34	27 (96)	28
Thrombocytopenia	53 (49)	109	39 (56)	70	14 (36)	39
Lymphopenia	94 (86)	109	63 (95)	66	31 (72)	43
Outcomes						
Time in hospital, d						
Median (IQR)	8 (5-12)	170	7 (5-12)	112	8 (4-12)	58
1	3 (2)	173	2 (2)	112	1 (2)	61
2-7	81 (47)	173	54 (48)	112	27 (44)	61
8-14	52 (30)	173	31 (28)	112	21 (34)	61
≥15	35 (20)	173	23 (21)	112	12 (20)	61
ICU admission	115 (57)	201	60 (49)	122	55 (70)	79
Death	15 (7)	220	12 (10)	122	3 (3)	98

Abbreviations: BNP, B-type natriuretic peptide; CDC, Centers for Disease Control and Prevention; COVID-19, coronavirus-19 disease; ICU, intensive care unit; IQR, interquartile range; MIS-A, multisystem inflammatory syndrome in adults; NT-proBNP, N-terminal proBNP; RT-PCR, reverse transcriptase–polymerase chain reaction.

^a^ Includes multiracial, Native American/Alaska Native, and Native Hawaiian/Pacific Islander.

^b^ Fever was 38 °C or higher for patients reported to the CDC and subjective or 38 °C or higher for patients reported in the literature.

^c^ Includes missing serologic results or serologic testing not performed.

^d^ Refers to tocilizumab, an interleukin 6 receptor inhibitor, and anakinra, an interleukin 1 receptor antagonist.

^e^ Thrombocytopenia was defined as a platelet count less than 150 000/μL; lymphopenia, as a white blood cell count less than 3000/μL.

Most patients with MIS-A presented with fever (197 of 205 [96%]), hypotension (133 of 220 [60%]), cardiac dysfunction (114 of 210 [54%]), shortness of breath (102 of 198 [52%]), and/or diarrhea (102 of 197 [52%]). The organ systems most affected were hematologic (184 of 200 [92%]), cardiovascular (193 of 221 [87%]), gastrointestinal tract (182 of 218 [83%]), and respiratory (159 of 215 [74%]); a median of 5 (IQR, 4-6) organ systems was involved. Myocarditis was reported in 61 of 205 patients (30%); 44 of 175 (25%) had pericardial effusion (Table). Ten of 94 patients with MIS-A reported in the literature (11%) presented with Kawasaki disease (median age, 37 [IQR, 33-44] years). None of the patients with MIS-A reported to the CDC met the criteria for Kawasaki disease. Nine of 195 patients (5%) experienced arterial or venous thrombosis. One patient reported in the literature^16^ had severe mononeuritis multiplex, affecting the right median and facial nerves and both ulnar, tibial, peroneal, and sural nerves, in addition to myocarditis and cardiogenic shock.

Most patients had an elevated D-dimer level (138 of 151 [91%]) and/or lymphopenia (94 of 109 [86%]). Most patients (176 of 195 [90%]) had elevated markers of coagulopathy and/or inflammation. Among patients with laboratory investigations, each had elevated levels of at least 1 of the following: interleukin 6 (61 of 62 [98%]), ferritin (150 of 165 [91%]), fibrinogen (93 of 102 [91%]), C-reactive protein (176 of 195 [90%]), B-type natriuretic peptide (BNP) (56 of 76 [74%]), and N-terminal proBNP (NT-proBNP) (53 of 59 [90%]) (Table). For the subset of case reports to the CDC with available data, the median peak values of inflammatory markers were 86 (IQR, 35-229) pg/mL for interleukin 6 (reference range, ≤1.8 pg/mL [n = 34]), 1029 (IQR, 422-3094) ng/mL for ferritin (reference range, 12-300 ng/mL for men and 12-150 ng/mL for women [n = 93]), 24 (IQR, 19-34) mg/dL for C-reactive protein (reference range, 0-10 mg/dL [n = 105]), 624 (IQR, 473-722) mg/dL for fibrinogen (reference range, 200-400 mg/dL [n = 77]), 271 (IQR, 163-900) for BNP (reference range, <100 pg/mL [n = 43]), and 2219 (IQR, 318-9491) ng/L for NT-proBNP (reference range, <125 ng/L [n = 36]).

Of 211 patients with available data, 207 (98%) had laboratory evidence of current or past SARS-CoV-2 infection; 188 of 194 (97%) had positive serologic and/or RT-PCR test results. Both RT-PCR and serologic test results were positive in 62 of 192 patients (32%) during the hospitalization. Of note, 139 of 194 patients (72%) were seropositive; 49 of 194 (25%) had positive RT-PCR results only, and 77 of 194 (40%) had positive serologic results only.

Treatment of MIS-A included anticoagulants (eg, heparin, enoxaparin) in 110 of 193 patients (57%), corticosteroids (eg, dexamethasone) in 152 of 205 (74%), intravenous immunoglobulin in 112 of 205 (55%), and immune modulators (eg, tocilizumab) in 42 of 203 (21%). The median hospital stay was 8 (IQR, 5-12) days. Patients with MIS-A were severely ill: 110 of 214 (51%) had shock/hypotension requiring vasoactive medications, 115 of 201 (57%) were admitted to the intensive care unit, 101 of 213 (47%) required respiratory support (of whom 53 [52%] needed mechanical ventilation), and 15 of 220 (7%) died.

Compared with patients with MIS-C who were younger than 18 years (n = 3639), patients with MIS-A (n = 221) were more likely to report previous COVID-19 (102 of 149 [68%] vs 826 of 2858 [29%]; *P* < .001) and to present with myocarditis (61 of 205 [30%] vs 543 of 3639 [15%]; *P* < .001), cardiac dysfunction (114 of 210 [54%] vs 975 of 3362 [29%]; *P* < .001), and arterial thrombosis, pulmonary embolism, and/or deep venous thrombosis (9 of 195 [5%] vs 24 of 3639 [1%]; *P* < .001). Patients with MIS-C were more likely to have dermatologic and mucocutaneous manifestations (2755 of 3639 [76%] vs 100 of 218 [46%]; *P* < .001) and to receive intravenous immunoglobulin (3121 of 3639 [86%] vs 112 of 205 [55%]; *P* < .001) compared with patients with MIS-A. Patients with MIS-A had longer hospital stays (median, 8 [IQR, 5-12] days [n = 180] vs 5 [IQR, 4-8] days [n = 3639]; *P* < .001), and higher proportions needed ventilation (53 of 213 [25%] vs 338 of 3639 [9%]; *P* < .001) and died (15 of 220 [7%] vs 27 of 3639 [1%]; *P* < .001) compared with patients with MIS-C.

## Discussion

The true incidence of MIS-A is unknown, but it appears to be rare. In our review, most patients with MIS-A were young (aged 19-34 years), male, and either non-Hispanic Black or Hispanic persons. Clinicians should consider a diagnosis of MIS-A among persons with hyperinflammatory illness and severe extrapulmonary multiorgan dysfunction, particularly cardiovascular, occurring within 2 to 5 weeks of antecedent COVID-19 or exposure to a person with diagnosed COVID-19. Because data on known SARS-CoV-2 infection or exposure are not always available at the time of hospital admission, clinicians should maintain a high index of suspicion for MIS-A among patients in whom a history of illness is not known. These patients should undergo evaluation for current or previous SARS-CoV-2 infection (by RT-PCR, rapid antigen tests, or serologic tests for antibodies, including measuring titers) and for severe inflammation and/or coagulopathy (eg, elevated C-reactive protein, ferritin, interleukin 6, or D-dimer levels). Interim recommendations for MIS-C treatment include corticosteroids, intravenous immunoglobulin, or possibly other immunomodulators.^73^ In this systematic review, clinicians reported using these treatments for MIS-A as well. However, further investigation of MIS-A is needed to strengthen diagnostic criteria and understand its association with postacute sequelae of SARS-CoV-2 infection and to identify effective treatments.

Multisystem inflammatory syndrome seems to have different phenotypes across the age spectrum, and persons older than 18 years are more likely to report previous COVID-19.^3^ Given that more than 50% of patients with MIS-A came from the MIS-C surveillance system, there is an inherent bias toward reporting among younger age groups. However, the other patients drawn from the literature were quite young as well (median age, 33 [IQR, 24-45] years); it is unclear whether this observation is a result of detection bias or whether MIS-A occurs mainly in younger adults. In addition, some cases may represent acute biphasic COVID-19, particularly among persons with delayed severe pulmonary manifestations. Large data registries and clinical cohorts are needed to further define and distinguish between these clinical entities as well as mitigate the selection bias.

Although extrapulmonary manifestations and elevated laboratory markers of coagulopathy and inflammation are also seen in severe COVID-19,^74^ MIS-A appears to be distinct in that it presents as a postacute, postinfectious illness, often after a period of recovery, and is heralded by the onset of new symptoms. However, efforts are needed to distinguish it from biphasic acute COVID-19. Using both RT-PCR and serologic testing for antibodies may aid in the diagnosis of MIS-A, especially among persons who had asymptomatic acute COVID-19, because IgG antibodies become detectable 3 to 4 weeks after SARS-CoV-2 infection,^75^ at the time when MIS-A commonly presents (median, 28 [IQR, 20-36] days). In addition, owing to comorbidities, some individuals may be delayed in mounting an antibody response and have persistently positive SARS-CoV-2 RNA. As SARS-CoV-2 vaccination programs are implemented, antibody assays that distinguish between antibody response to natural infection, which produces antibodies to the nucleocapsid protein, and vaccination, which produces antibodies to the spike protein of SARS-CoV-2, have been authorized for use in the US.^76^

The apparent increased occurrence of MIS-A among men and some minority populations requires further study. Sex differences in COVID-19 severe outcomes and cardiovascular disease have been reported, suggesting the possibility that biological and/or behavioral differences predispose men to both illnesses.^77^ In the US, it is widely recognized that minority populations are at higher risk of COVID-19, which is thought to be largely owing to long-standing health and social inequities.^78,79^ In the UK, compared with persons of White ethnicities, persons of Bangladeshi, Pakistani, Indian, and mixed ethnicities also have a significantly increased risk of COVID-19–related death.^80^ Further investigation is warranted to better understand and address both socioeconomic factors and potential biological factors that predispose minority populations to severe outcomes and consequences of COVID-19 globally.

**Figure 2.**
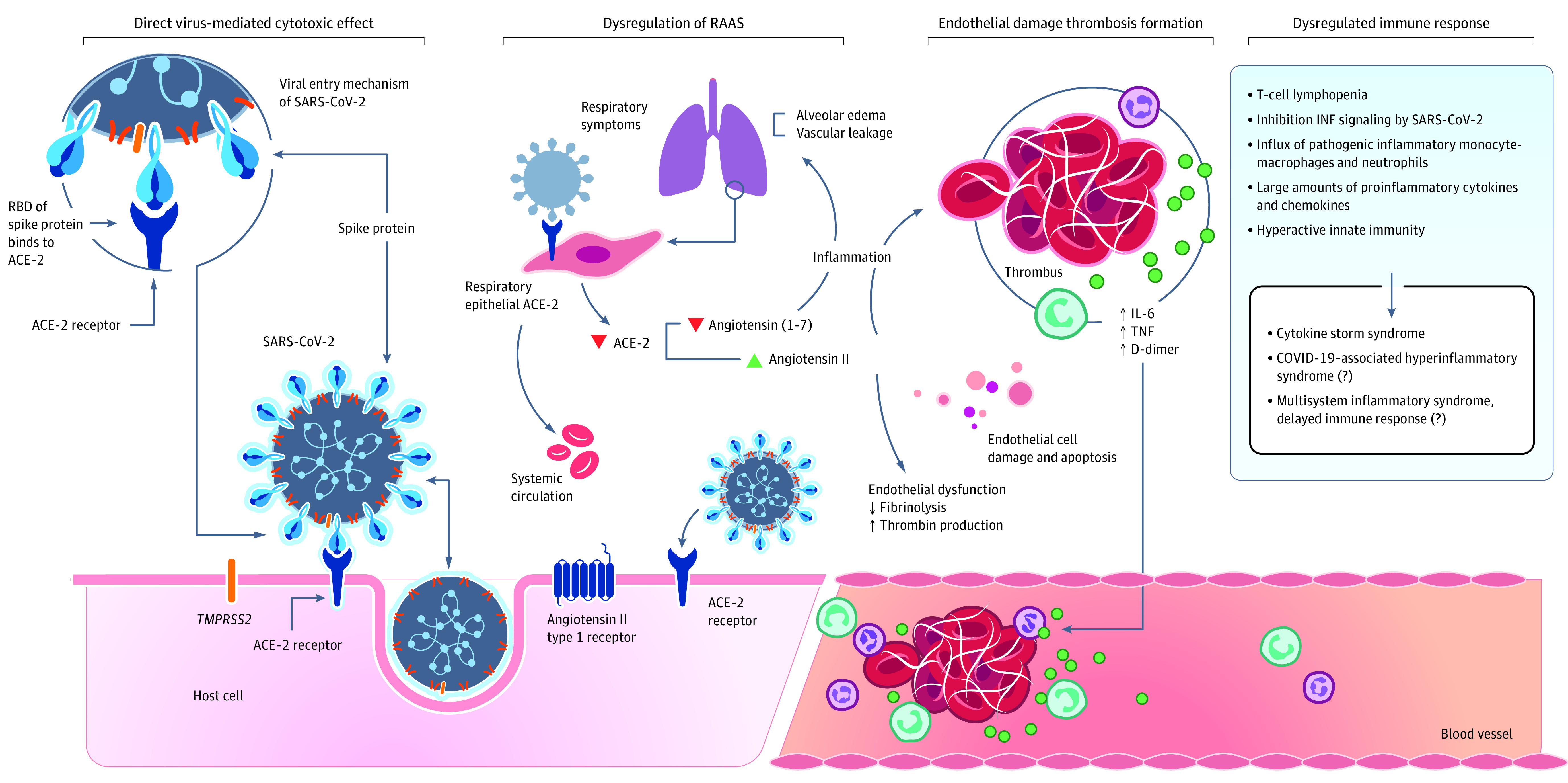
Plausible Mechanisms for the Pathogenesis of COVID-19 SARS-CoV-2 enters host cells through interaction of its spike protein with angiotensin-converting enzyme 2 (ACE-2) receptors. Plausible mechanisms of injury include direct virus-mediated cytotoxic effects; dysregulation of the renin-angiotensin-aldosterone system (RAAS) resulting from downregulation of ACE-2 related to viral entry, subsequent increase in angiotensin II levels, and potential decrease in angiotensin 1-7 causing viral-induced inflammation; endothelial damage and thrombus formation; and dysregulation of the immune response with hyperinflammation caused by inhibition of interferon (INF), depletion of T lymphocytes, and production of proinflammatory cytokines. IL-6 indicates interleukin 6; RBD, receptor-binding domain; and TNF, tumor necrosis factor.

COVID-19 causes both significant respiratory and extrapulmonary pathology. Because the SARS-CoV-2 spike protein has an affinity for angiotensin-converting enzyme 2 receptors, which facilitate entry of the virus into cells, extrapulmonary manifestations are often noted in tissues that express angiotensin-converting enzyme 2 receptors.^74^ Plausible mechanisms of injury include direct virus-mediated cytotoxic effects; dysregulation of the renin-angiotensin-aldosterone system resulting from downregulation of angiotensin-converting enzyme 2 and causing viral-induced inflammation; endothelial damage and thromboinflammation; and dysregulation of the immune response with hyperinflammation caused by inhibition of interferon, depletion of T lymphocytes, and production of proinflammatory cytokines (Figure 2).^75,81^ The hyperinflammatory syndrome of COVID-19 shares similarities with cytokine release syndromes.^82^ Criteria to identify COVID-19–associated hyperinflammatory syndrome have been proposed and are being validated.^82^ The relationship between MIS-A and COVID-19–associated hyperinflammatory syndrome is not yet clear. For example, lymphopenia is an early indicator for severe COVID-19 with COVID-19–associated hyperinflammatory syndrome, but it is unclear whether the same is true for MIS-A, because this has not yet been studied.

**Figure 3.**
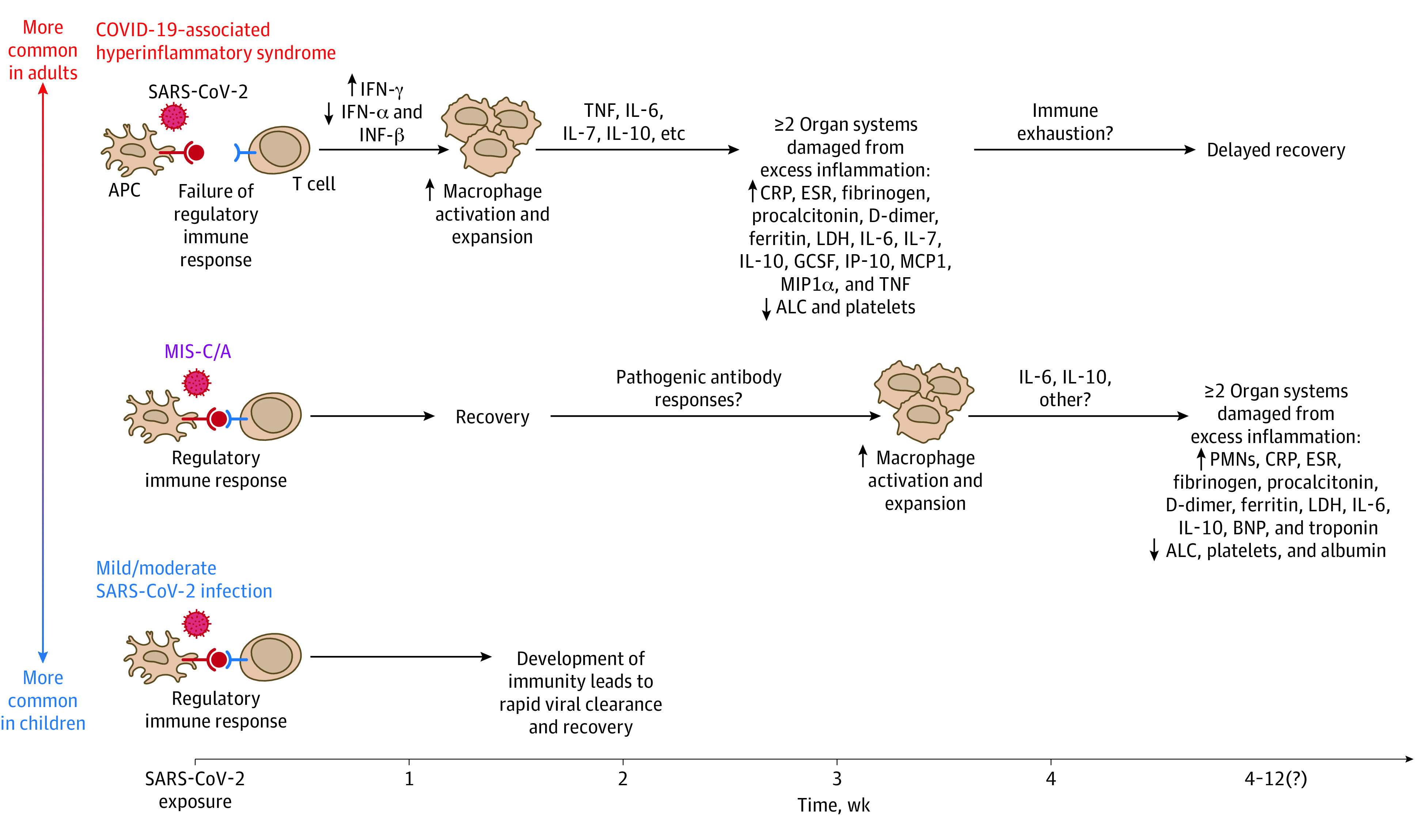
Potential Mechanisms of Inflammatory Syndromes Associated With SARS-CoV-2 SARS-CoV-2 can trigger a range of inflammatory syndromes across the age spectrum. Compared with children, adults—particularly those with certain preexisting proinflammatory comorbidities—are more likely to develop acute COVID-19–associated hyperinflammatory syndrome within 1 to 2 weeks of exposure to SARS-CoV-2. COVID-19–associated hyperinflammatory syndrome begins with failure of the regulatory immune response to SARS-CoV-2, including abnormal interferon (INF) production that drives macrophage hyperactivation. This results in inflammatory cytokine cascades and causes significant damage to multiple organ systems. In contrast, children are more likely to have asymptomatic or mild acute SARS-CoV-2 infection without sequelae. The reason(s) why children do not commonly develop acute COVID-19–associated hyperinflammatory syndrome remains unknown. However, both children and adults can develop a multisystem inflammatory syndrome (MIS-C/A) of unclear etiology weeks after initial asymptomatic or mild SARS-CoV-2 infection. The precise cause of MIS-C/A remains unclear but may be due to development of abnormal antibody responses that drive systemic hyperinflammation.^81^ ALC indicates absolute lymphocyte count; APC, antigen-presenting cell; BNP, B-type natriuretic peptide; CRP, C-reactive protein; ESR, erythrocyte sedimentation rate; GCSF, granulocyte colony-stimulating factor; IL, interleukin; IP-10, human interferon-inducible protein 10; LDH, lactate dehydrogenase; MCP, monocyte chemotactic protein; MIP, macrophage inflammatory protein; PMN, polymorphonuclear leukocyte; and TNF, tumor necrosis factor. Reproduced with permission from Prathit Arun Kulkarni (Weatherhead et al^83^) on December 20, 2020.

Multisystem inflammatory syndrome among adults is proposed to result from a delayed, dysregulated immune response (Figure 3).^83^ Multisystem inflammatory syndrome among children has been postulated to involve a dysregulated immune response with reduced neutralizing antibody levels and diminished functional capacity leading to low-level persistent infection in extrapulmonary tissues.^84^ In addition, among children with MIS, autoreactive antibodies have been recently identified that may promote anomalous immune responses promoting inflammation.^84^ The same processes may occur in adults with MIS^82^; however, in adults, the balance between antiviral and proinflammatory responses may be negatively influenced by age, leading to hyperinflammation.^85^ Immunosenescence (aging of the immune cells) and inflammation due to aging, as well as immunosuppression due to comorbidities and medications, may be factors that complicate or obscure the presentation of MIS-A.^85^ This may explain why the patients in our systematic review were mostly young and middle-aged adults with few comorbidities. It is likely that MIS-A occurs in older adults but that the presentation may be more complex and challenging to diagnose. It is also possible that MIS-A may be a late sequela of acute COVID-19. Further research is needed to understand the immunopathogenesis of MIS-A; immunotyping and testing specimens for a variety of immune markers such as interleukins and tumor necrosis factors may identify pathognomonic markers. Studies of immunopathology should help to understand the pathophysiology of MIS-A and whether endotheliitis or autoimmune and/or other mechanisms are responsible, in association with other hyperinflammatory phenotypes and other consequences of COVID-19.

### Limitations

Some limitations of this study should be noted. This study is primarily descriptive and combines data from multiple sources, which might result in an inherent reporting bias. We note above that using data from the MIS-C surveillance system may have contributed to selection bias and thus a propensity toward reporting cases in the younger group. However, selection bias might also affect the distribution of other characteristics such as (1) the time from COVID-19 diagnosis to MIS-A, because younger individuals are less likely to report acute COVID-19 or symptomatic SARS-CoV-2 infection, and bias may arise, for example, if shorter times would be considered biphasic acute COVID-19; (2) intensive care unit admission, which may reflect severe COVID-19; and (3) comorbidities, which may influence whether MIS-A is considered or reported given the unusual presentation similar to that of patients with cancer.^60^ Another limitation is our inability to report specifics of SARS-CoV-2 antibody testing (eg, brand of test performed, IgM vs IgG, and titers), given the limited availability and lack of consistent use during 2020, as well as other laboratory parameters that may be important to the diagnosis of MIS-A.

## Conclusions

It is important for the clinical and public health community to suspect and identify MIS-A, a delayed immunologic response to SARS-CoV-2 infection in adults with hyperinflammation, by exercising clinical acumen and considering empirical treatment to reduce related morbidity and mortality. We have summarized the current evidence and knowledge about MIS-A. Moving forward, improvements in our understanding of MIS-A will require engagement across public health, translational research, and health care systems. In particular, prospective research should systematically screen for MIS-A to reveal a more accurate representation of this clinical entity. Currently, the best way to prevent COVID-19 and its dire outcomes, including hyperinflammatory syndromes such as MIS-A, is to prevent SARS-CoV-2 infection and transmission.

## Notes

Supplement. **eAppendix.** US MIS-C Surveillance Case Report Form **eTable 1.** Literature Review Search Strategy **eTable 2.** Quality Rating Scheme for Studies (Modified From the Oxford Centre for Evidence-Based Medicine for Ratings of Individual Studies)
