# Supplementary material for: Clinical Characteristics of Multisystem Inflammatory Syndrome in Adults: A Systematic Review
Source: JAMA Netw Open. 2021 Sep 22;4(9):e2126456. doi: 10.1001/jamanetworkopen.2021.26456 (PMC8459192; doi:10.1001/jamanetworkopen.2021.26456)
Supplement: Supplement. — eAppendix. US MIS-C Surveillance Case Report Form eTable 1. Literature Review Search Strategy eTable 2. Quality Rating Scheme for Studies (Modified From the Oxford Centre for Evidence-Based Medicine for Ratings of Individual Studies) [file jamanetwopen-e2126456-s001.pdf]

Supplementary Online Content

Patel P, DeCuir J, Abrams J, Campbell AP, Godfred-Cato S, Belay ED. Clinical characteristics of multisystem inflammatory syndrome in adults: a systematic review. *JAMA Netw Open*. 2021;4(9):e2126456. doi:10.1001/jamanetworkopen.2021.26456

eAppendix. US MIS-C Surveillance Case Report Form

eTable 1. Literature Review Search Strategy

eTable 2. Quality Rating Scheme for Studies (Modified From the Oxford Centre for Evidence-Based Medicine for Ratings of Individual Studies)

This supplementary material has been provided by the authors to give readers additional information about their work.

©2021 Patel P et al. *JAMA Network Open*.

eAppendix. US MIS-C Surveillance Case Report Form

U.S. DEPARTMENT OF HEALTH AND HUMAN SERVICES
CENTERS FOR DISEASE CONTROL AND PREVENTION
ATLANTA, GA 30329

Multisystem Inflammatory Syndrome Associated with COVID-19 Case Report Form

MIS ID *(REQUIRED)* : ____________________ **Health Department ID:** ________________

NNDSS ID *(local_record_id/case id)*: ________________ **Tools for CRF data submission to supplement NNDSS case notification/data:** ○ DCIPHER ○ RedCap

Abstractor name: ________________________________ **NCOV ID** (If available): ____________ **Date of abstraction:** __________

SECTION 1 – INCLUSION CRITERIA

1.1 ☐ Age <21, AND

1.2 ☐ Fever >38.0°C for ≥24 hours, or report of subjective fever lasting ≥24 hours, AND

1.3 ☐ Laboratory markers of inflammation (including, but not limited to one or more; an elevated C-reactive protein (CRP), erythrocyte sedimentation rate (ESR), fibrinogen, procalcitonin, d-dimer, ferritin, lactic acid dehydrogenase (LDH), or interleukin 6 (IL-6), elevated neutrophils, reduced lymphocytes and low albumin, AND

1.4 ☐ Evidence of clinically severe illness requiring hospitalization, with multisystem (≥2) organ involvement *(check all applicable below)*: AND

- **1.4.1** ☐ Cardiac (e.g. shock, elevated troponin, BNP, abnormal echocardiogram, arrhythmia)
- **1.4.2** ☐ Renal (e.g. acute kidney injury or renal failure)
- **1.4.3** ☐ Respiratory (e.g. pneumonia, ARDS, pulmonary embolism)
- **1.4.4** ☐ Hematologic (e.g. elevated D-dimers, thrombophilia, or thrombocytopenia)
- **1.4.5** ☐ Gastrointestinal (e.g. elevated bilirubin, elevated liver enzymes, or diarrhea)
- **1.4.6** ☐ Dermatologic, (e.g. rash, mucocutaneous lesions)
- **1.4.7** ☐ Neurological, (e.g. CVA, aseptic meningitis, encephalopathy)

1.5 ☐ No alternative plausible diagnosis; AND

1.6 ☐ Positive for current or recent SARS-COV-2 infection by (check all applicable below): OR

- **1.6.1** ☐ RT-PCR
- **1.6.2** ☐ Serology
- **1.6.3** ☐ Antigen test

1.7 ☐ COVID-19 exposure within the 4 weeks prior to the onset of symptoms

- **1.7.1** If yes, date of first exposure within the 4 weeks prior : *(MM/DD/YYYY)*: ____________ ☐ Unknown

SECTION 2 – PATIENT DEMOGRAPHICS

2.1 **State of Residence:** ______________

2.2 **Patient zip code**/postal code *(primary residence)*: ________

2.3 **Date of birth** *(MM/DD/YYYY)*: ____________

2.4 **Sex:** ○ Male ○ Female

2.5 **Ethnicity:** ○ Hispanic or Latino ○ Not Hispanic or Latino ○ Refused or Unknown

2.6 **Race** *(mark all that apply, selecting more than one option as necessary)*:

- **2.6.1** ☐ White
- **2.6.2** ☐ Black or African American
- **2.6.3** ☐ American Indian
- **2.6.4** ☐ Alaska Native or Aboriginal Canadian
- **2.6.5** ☐ Native Hawaiian
- **2.6.6** ☐ Other Pacific Islander
- **2.6.7** ☐ Asian
- **2.6.8** ☐ Other
- **2.6.9** ☐ Refused or Don't know

2.7 **Height:** ________ inches

2.8 **Weight:** ________ lbs

2.9 **BMI:** ________

Comorbidities:

2.10.1	Immunosuppressive disorder/malignancy	○ Yes	○ No
2.10.2	Obesity	○ Yes	○ No
2. 10.3	Type 1 diabetes	○ Yes	○ No
2. 10.4	Type 2 diabetes	○ Yes	○ No
2. 10.5	Seizures	○ Yes	○ No
2. 10.6	Congenital heart disease	○ Yes	○ No
2. 10.7	Sickle cell disease	○ Yes	○ No
2. 10.8	Chronic lung disease	○ Yes	○ No
2. 10.9	Other congenital malformations	○ Yes	○ No
2. 10.10	Other *(specify)*: ______________________		

2.11 Hospital admission date *(MM/DD/YYYY)*: ____________

2.11.1 Number of days in the hospital: ________

2.12 If admitted to the ICU, admission date *(MM/DD/YYYY)*: ____________

2.12.1 Number of days in the ICU: ________

2.13 **Patient outcome:** ○ Died ○ Discharged ○ Still admitted

2.13.2 Hospital discharge or death date *(MM/DD/YYYY)*: ____________

©2021 Patel P et al. *JAMA Network Open*.

SECTION 3 – CLINICAL SIGNS AND SYMPTOMS

3.1 Did the patient have preceding COVID-like illness? ◯ Yes ◯ No

3.1.1 Date of symptom onset *(MM/DD/YYYY)*: ____________

3.2 Date of symptom onset of MIS *(MM/DD/YYYY)*: ____________

3.3. Fever ≥ 38.0°C: ◯ Yes ◯ No

3.3.1 Date of fever onset *(MM/DD/YYYY)*: ____________

3.3.2 Highest Temperature: _____ °C

3.3.3 Number of days febrile: _____

Signs and symptoms *during present illness*

**3.4.1**	**Cardiac**		
3.4.1.1	Shock	◯ Yes	◯ No
3.4.1.2	Elevated troponin	◯ Yes	◯ No
3.4.1.3	Elevated BNP or NT-proBNP	◯ Yes	◯ No
**3.4.2**	**Renal**		
3.4.2.1	Acute kidney injury	◯ Yes	◯ No
3.4.2.2	Renal failure	◯ Yes	◯ No
**3.4.3**	**Respiratory**		
3.4.3.1	Cough	◯ Yes	◯ No
3.4.3.2	Shortness of breath	◯ Yes	◯ No
3.4.3.3	Chest pain/tightness	◯ Yes	◯ No
3.4.3.4	Pneumonia	◯ Yes	◯ No
3.4.3.5	ARDS	◯ Yes	◯ No
3.4.3.6	Pulmonary embolism	◯ Yes	◯ No
**3.4.4**	**Hematologic**		
3.4.4.1	Elevated D-dimers	◯ Yes	◯ No
3.4.4.2	Thrombophilia	◯ Yes	◯ No
3.4.4.3	Thrombocytopenia	◯ Yes	◯ No
**3.4.5**	**Gastrointestinal**		
3.4.5.1	Abdominal pain	◯ Yes	◯ No
3.4.5.2	Vomiting	◯ Yes	◯ No
3.4.5.3	Diarrhea	◯ Yes	◯ No
3.4.5.4	Elevated bilirubin	◯ Yes	◯ No
3.4.5.5	Elevated liver enzymes	◯ Yes	◯ No
**3.4.6**	**Dermatologic**		
3.4.6.1	Rash	◯ Yes	◯ No
3.4.6.2	Mucocutaneous lesions	◯ Yes	◯ No
**3.4.7**	**Neurological**		
3.4.7.1	Headache	◯ Yes	◯ No
3.4.7.2	Altered mental state	◯ Yes	◯ No
3.4.7.3	Syncope/near syncope	◯ Yes	◯ No
3.4.7.5	Meningitis	◯ Yes	◯ No
3.4.7.6	Encephalopathy	◯ Yes	◯ No
**3.4.8**	**Other**		
3.4.8.1	Neck pain	◯ Yes	◯ No
3.4.8.2	Myalgia	◯ Yes	◯ No
3.4.8.3	Conjunctival injection	◯ Yes	◯ No
3.4.8.4	Periorbital edema	◯ Yes	◯ No
3.4.8.5	Cervical lymphadenopathy >1.5 cm diameter	◯ Yes	◯ No

SECTION 4 – COMPLICATIONS

**4.1**	**Arrhythmia**	◯ Yes	◯ No
	If yes:		
4.1.1	Ventricular arrhythmia:	◯ Yes	◯ No
4.1.2	Supraventricular arrhythmia:	◯ Yes	◯ No
4.1.3	Other arrhythmia *(specify)*: ____________	◯ Yes	◯ No
4.2	Congestive heart failure	◯ Yes	◯ No
4.3	Myocarditis	◯ Yes	◯ No
4.4	Pericarditis	◯ Yes	◯ No
4.5	Liver failure	◯ Yes	◯ No
4.6	Deep vein thrombosis or PE	◯ Yes	◯ No
4.7	ARDS	◯ Yes	◯ No
4.8	Pneumonia	◯ Yes	◯ No
4.9	CVA or stroke	◯ Yes	◯ No
4.10	Encephalitis or aseptic meningitis	◯ Yes	◯ No
4.11	Shock	◯ Yes	◯ No
4.12	Hypotension	◯ Yes	◯ No

SECTION 5 – TREATMENTS

5.1	Low flow nasal cannula	◯ Yes	◯ No
5.2	High flow nasal cannula	◯ Yes	◯ No
5.3	Non-invasive ventilation	◯ Yes	◯ No
5.4	Intubation	◯ Yes	◯ No
5.5	Mechanical ventilation	◯ Yes	◯ No
5.6	ECMO	◯ Yes	◯ No
5.7	Vasoactive medications (e.g. epinephrine, milrinone, norepinephrine, or vasopressin) *(specify)*: ____________	◯ Yes	◯ No
5.8	Steroids	◯ Yes	◯ No
5.9	Immune modulators (e.g. anakinra, tocilizumab) *(specify)*: ____________	◯ Yes	◯ No
5.10	Antiplatelets (e.g. aspirin, clopidogrel) *(specify)*: ____________	◯ Yes	◯ No
5.11	Anticoagulation (e.g. heparin, enoxaparin, warfarin) *(specify)*: ____________	◯ Yes	◯ No
5.12	Dialysis	◯ Yes	◯ No
5.13	First IVIG	◯ Yes	◯ No
5.14	Second IVIG	◯ Yes	◯ No

©2021 Patel P et al. *JAMA Network Open*.

SECTION 6 – STUDIES

6.1 Blood Test Results

6.1.1 Fibrinogen	Highest value: ____	units: ____	Low	Normal	High
6.1.2 CRP	Highest value: ____	units: ____	Low	Normal	High
6.1.3 Ferritin	Highest value: ____	units: ____	Low	Normal	High
6.1.4 Troponin	Highest value: ____	units: ____	Low	Normal	High
6.1.5 BNP	Highest value: ____	units: ____	Low	Normal	High
6.1.6 NT-proBNP	Highest value: ____	units: ____	Low	Normal	High
6.1.7 D-dimer	Highest value: ____	units: ____	Low	Normal	High
6.1.8 IL-6	Highest value: ____	units: ____	Low	Normal	High

6.1.9 Serum White Blood Count	Highest value: ____	Lowest value : ____	units: ____
6.1.10 Platelets	Highest value: ____	Lowest value : ____	units: ____
6.1.11 Neutrophils	Highest value: ____	Lowest value : ____	units: ____
6.1.12 Lymphocytes	Highest value: ____	Lowest value : ____	units: ____
6.1.13 Bands	Highest value: ____	Lowest value : ____	units: ____

6.2 CSF Studies

6.2.1 White blood count	Highest value: ____	Lowest value : ____	units: ____
6.2.2 Protein	Highest value: ____	Lowest value : ____	units: ____
6.2.3 Glucose	Highest value: ____	Lowest value : ____	units: ____

6.3 Urinalysis

6.3.1 Urine White blood count	Highest value : ____	Lowest value : ____	units: ____

6.4 Echocardiogram (check if seen on ANY echocardiogram)

- 6.4.1 ☐ Not done
- 6.4.2 ☐ Normal results
- 6.4.3 ☐ Coronary artery aneurysms
  - 6.4.3.1 Max coronary artery Z-score: ____
- 6.4.4 ☐ Coronary artery dilatation
- 6.4.5 ☐ Cardiac dysfunction (decreased function), specify type:
  - 6.4.5.1 ☐ left ventricular dysfunction
  - 6.4.5.2 ☐ right ventricular dysfunction
- 6.4.6 ☐ Pericardial effusion
- 6.4.7 ☐ Pleural effusion
- 6.4.8 ☐ Mitral regurgitation, specify type: ○ mild ○ moderate ○ severe
- 6.4.9 ☐ Other *(specify)*: ____

6.5 Date of first test showing coronary artery aneurysm or dilatation *(MM/DD/YYYY)*: ____

6.6 Abdominal imaging ☐ Ultrasound ☐ CT ☐ Not done

- 6.6.1 ☐ Normal
- 6.6.2 ☐ Mesenteric lymphadenopathy
- 6.6.3 ☐ Free fluid
- 6.6.4 ☐ Other *(specify)*: ____

6.7 Chest imaging ☐ Chest x-ray ☐ CT ☐ Not done

- 6.7.1 ☐ Normal
- 6.7.2 ☐ Pneumonia
- 6.7.3 ☐ Atelectasis
- 6.7.4 ☐ Pleural effusion
- 6.7.5 ☐ Other *(specify)*: ____

SARS-COV-2 testing

6.8 RT-PCR: ○ Positive ○ Negative ○ Not done
6.8.1 If performed, date *(MM/DD/YYYY)*: ____

6.9 Antigen: ○ Positive ○ Negative ○ Not done
6.9.1 If performed, date *(MM/DD/YYYY)*: ____

6.10 IgG: ○ Positive ○ Negative ○ Not done
6.10.1 If performed, date *(MM/DD/YYYY)*: ____

6.11 IgM: ○ Positive ○ Negative ○ Not done
6.11.1 If performed, date *(MM/DD/YYYY)*: ____

6.12 IgA: ○ Positive ○ Negative ○ Not done
6.12.1 If performed, date *(MM/DD/YYYY)*: ____

©2021 Patel P et al. *JAMA Network Open*.

eTable 1. Literature Review Search Strategy

Search Query: We would like to summarize recent reports of multisystem inflammatory syndrome in adults also reported as Kawaski-like illness in adults.

Search Strategy:

Database	Strategy	Records 12/14/2020	Records 05/25/2021
**Medline (OVID) 1946-**	((Multisystem* OR multi-system*) ADJ5 inflammat*) OR severe inflammation OR (multi* ADJ5 organ ADJ5 dysfunction) OR (multi* ADJ5 system ADJ5 dysfuntion) OR (multi* ADJ5 organ ADJ5 failure) OR (multi* ADJ5 system ADJ5 failure) OR MIS-A OR Kawasaki OR swelling OR (inflammat* AND shock) OR (inflammat* AND hypotension) OR (inflammat* AND cardiogenic) OR (inflammat* ADJ5 cardiac) OR cardiogenic shock OR vasodilatory shock OR pauci-inflammat* OR (inflammat* ADJ5 (myocardi* OR stroke OR thrombo* OR pericardi* OR valvulitis OR coronary OR arrythmia* OR liver))<br>AND<br>Exp adult/ OR (adult* OR senior* OR aged OR elder* OR man OR woman OR men OR women).ti,ab.<br>AND<br>Limit COVID-19 (validated filter)<br>(202012* OR 2021*).dt	273	353
**Embase (OVID) 1988-**	((Multisystem* OR multi-system*) ADJ5 inflammat*) OR severe inflammation OR (multi* ADJ5 organ ADJ5 dysfunction) OR (multi* ADJ5 system ADJ5 dysfuntion) OR (multi* ADJ5 organ ADJ5 failure) OR (multi* ADJ5 system ADJ5 failure) OR MIS-A OR Kawasaki OR swelling OR (inflammat* AND shock) OR (inflammat* AND hypotension) OR (inflammat* AND cardiogenic) OR (inflammat* ADJ5 cardiac) OR cardiogenic shock OR vasodilatory shock OR pauci-inflammat* OR (inflammat* ADJ5 (myocardi* OR stroke OR thrombo* OR pericardi* OR valvulitis OR coronary OR arrythmia* OR liver))<br>AND<br>Exp adult/ OR (adult* OR senior* OR aged OR elder* OR man OR woman OR men OR women).ti,ab.<br>AND<br>Limit COVID-19 (validated filter)<br>(202012* OR 2021*).dc	885<br>-237 duplicates<br>=648 unique items	1431<br>-252 duplicates<br>=1179 unique items

©2021 Patel P et al. *JAMA Network Open*.

	Not pubmed/medline		
**Global Health (OVID)**	(novel coronavir* OR novel corona virus* OR 2019 coronavirus OR coronavirus disease OR coronavirus 2019 OR betacoronavir* OR covid19 OR covid 19 OR nCoV OR novel CoV OR CoV 2 OR CoV2 OR sarscov2 OR sars-cov OR sarscov OR 2019nCoV OR 2019-nCoV OR wuhan virus*) OR (((wuhan OR hubei OR huanan) AND (severe acute respiratory OR pneumonia*) AND outbreak*) AND 2020*.up) OR ((coronavirus OR pandemic).mp AND 2020*.up)<br><br>AND<br><br>((Multisystem* OR multi-system*) ADJ5 inflammat*) OR severe inflammation OR (multi* ADJ5 organ ADJ5 dysfunction) OR (multi* ADJ5 system ADJ5 dysfuntion) OR (multi* ADJ5 organ ADJ5 failure) OR (multi* ADJ5 system ADJ5 failure) OR MIS-A OR Kawasaki OR swelling OR (inflammat* AND shock) OR (inflammat* AND hypotension) OR (inflammat* AND cardiogenic) OR (inflammat* ADJ5 cardiac) OR cardiogenic shock OR vasodilatory shock OR pauci-inflammat* OR (inflammat* ADJ5 (myocardi* OR stroke OR thrombo* OR pericardi* OR valvulitis OR coronary OR arrythmia* OR liver))<br><br>AND<br><br>Exp adult/ OR (adult* OR senior* OR aged OR elder* OR man OR woman OR men OR women).ti,ab.<br><br>AND<br><br>(202012* OR 2021*).up	32<br><br>-25 duplicates<br><br>=7 unique items	118<br><br>-54 duplicates<br><br>=64 unique items
**CAB Abstracts (OVID)**	(novel coronavir* OR novel corona virus* OR 2019 coronavirus OR coronavirus disease OR coronavirus 2019 OR betacoronavir* OR covid19 OR covid 19 OR nCoV OR novel CoV OR CoV 2 OR CoV2 OR sarscov2 OR sars-cov OR sarscov OR 2019nCoV OR 2019-nCoV OR wuhan virus*) OR (((wuhan OR hubei OR huanan) AND (severe acute respiratory OR pneumonia*) AND outbreak*) AND 2020*.up) OR ((coronavirus OR pandemic).mp AND 2020*.up)<br><br>AND<br><br>((Multisystem* OR multi-system*) ADJ5 inflammat*) OR severe inflammation OR (multi* ADJ5 organ ADJ5 dysfunction) OR (multi* ADJ5 system ADJ5 dysfuntion) OR (multi* ADJ5 organ ADJ5 failure) OR (multi* ADJ5 system ADJ5 failure) OR MIS-A OR Kawasaki OR swelling OR (inflammat* AND shock) OR (inflammat* AND hypotension) OR (inflammat* AND cardiogenic) OR (inflammat* ADJ5 cardiac) OR cardiogenic shock OR vasodilatory shock OR pauci-inflammat* OR (inflammat* ADJ5 (myocardi* OR stroke OR thrombo* OR pericardi* OR valvulitis OR coronary OR arrythmia* OR liver))<br><br>AND	2<br><br>-2 duplicates<br><br>=0 unique items	14<br><br>-14 duplicates<br><br>=0 unique items

©2021 Patel P et al. *JAMA Network Open*.

	Exp adult/ OR (adult* OR senior* OR aged OR elder* OR man OR woman OR men OR women).ti,ab.<br>AND<br>(202012* OR 2021*).up		
**PsycInfo (OVID) 1987-**	(novel coronavir* OR novel corona virus* OR 2019 coronavirus OR coronavirus disease OR coronavirus 2019 OR betacoronavir* OR covid19 OR covid 19 OR nCoV OR novel CoV OR CoV 2 OR CoV2 OR sarscov2 OR sars-cov OR sarscov OR 2019nCoV OR 2019-nCoV OR wuhan virus*) OR (((wuhan OR hubei OR huanan) AND (severe acute respiratory OR pneumonia*) AND outbreak*) AND 2020*.up) OR ((coronavirus OR pandemic).mp AND 2020*.up)<br>AND<br>((Multisystem* OR multi-system*) ADJ5 inflammat*) OR severe inflammation OR (multi* ADJ5 organ ADJ5 dysfunction) OR (multi* ADJ5 system ADJ5 dysfuntion) OR (multi* ADJ5 organ ADJ5 failure) OR (multi* ADJ5 system ADJ5 failure) OR MIS-A OR Kawasaki OR swelling OR (inflammat* AND shock) OR (inflammat* AND hypotension) OR (inflammat* AND cardiogenic) OR (inflammat* ADJ5 cardiac) OR cardiogenic shock OR vasodilatory shock OR pauci-inflammat* OR (inflammat* ADJ5 (myocardi* OR stroke OR thrombo* OR pericardi* OR valvulitis OR coronary OR arrythmia* OR liver))<br>AND<br>(adult* OR senior* OR aged OR elder* OR man OR woman OR men OR women)<br>AND<br>(202012* OR 2021*).up	0	1<br>-0 duplicates<br>=1 unique items
**CINAHL (EbscoHost)**	("novel coronavir*" OR "novel corona virus*" OR "2019 coronavirus" OR betacoronavir* OR covid19 OR "covid 19" OR nCoV OR "novel CoV" OR "CoV 2" OR CoV2 OR sarscov2 OR sars-cov OR sarscov OR 2019nCoV OR 2019-nCoV OR "wuhan virus*") OR (((wuhan OR hubei OR huanan) AND ("severe acute respiratory" OR pneumonia*) AND outbreak*) AND PY 2020) OR ((coronavirus OR pandemic) AND PY 2020)<br>AND<br>((Multisystem* OR multi-system*) N5 inflammat*) OR "severe inflammation" OR (multi* N5 organ N5 dysfunction) OR (multi* N5 system N5 dysfuntion) OR (multi* N5 organ N5 failure) OR (multi* N5 system N5 failure) OR MIS-A OR Kawasaki OR swelling OR (inflammat* AND shock) OR (inflammat* AND hypotension) OR (inflammat* AND cardiogenic) OR (inflammat* N5 cardiac) OR "cardiogenic shock" OR "vasodilatory shock" OR pauci-inflammat*	83<br>-70 duplicates<br>=13 unique items	71<br>-43 duplicates<br>=28 unique items

©2021 Patel P et al. *JAMA Network Open*.

	AND<br>(MH adult+) OR (TI (adult* OR senior* OR aged OR elder* OR man OR woman OR men OR women)) OR (AB (adult* OR senior* OR aged OR elder* OR man OR woman OR men OR women))<br><br>Dec 2020 – May 2021		
**Academic Search Complete**	(“novel coronavir*” OR “novel corona virus*” OR “2019 coronavirus” OR betacoronavir* OR covid19 OR “covid 19” OR nCoV OR “novel CoV” OR “CoV 2” OR CoV2 OR sarscov2 OR sars-cov OR sarscov OR 2019nCoV OR 2019-nCoV OR “wuhan virus*”) OR (((wuhan OR hubei OR huanan) AND (“severe acute respiratory” OR pneumonia*) AND outbreak*) AND PY 2020) OR ((coronavirus OR pandemic) AND PY 2020)<br>AND<br>((Multisystem* OR multi-system*) N5 inflammat*) OR “severe inflammation” OR (multi* N5 organ N5 dysfunction) OR (multi* N5 system N5 dysfuntion) OR (multi* N5 organ N5 failure) OR (multi* N5 system N5 failure) OR MIS-A OR Kawasaki OR swelling OR (inflammat* AND shock) OR (inflammat* AND hypotension) OR (inflammat* AND cardiogenic) OR (inflammat* N5 cardiac) OR “cardiogenic shock” OR “vasodilatory shock” OR pauci-inflammat*<br>AND<br>(MH adult+) OR (TI (adult* OR senior* OR aged OR elder* OR man OR woman OR men OR women)) OR (AB (adult* OR senior* OR aged OR elder* OR man OR woman OR men OR women))<br><br>December 2020 – May 2021	64<br><br>-42 duplicates<br><br>=22 unique items	171<br><br>-97 duplicates<br><br>=74 unique items
**Scopus**	TITLE-ABS(“novel coronavir*” OR “novel corona virus*” OR “2019 coronavirus” OR betacoronavir* OR covid19 OR "covid 19" OR ncov OR "CoV 2" OR cov2 OR sarscov2 OR sars-cov OR sarscov OR 2019ncov OR 2019-nCoV OR "novel CoV" OR "wuhan virus") AND TITLE-ABS(((Multisystem* OR multi-system*) W/5 inflammat*) OR “severe inflammation” OR (multi* W/5 organ W/5 dysfunction) OR (multi* W/5 system W/5 dysfuntion) OR (multi* W/5 organ W/5 failure) OR (multi* W/5 system W/5 failure) OR MIS-A OR Kawasaki OR swelling OR (inflammat* AND shock) OR (inflammat* AND hypotension) OR (inflammat* AND cardiogenic) OR (inflammat* W/5 cardiac) OR “cardiogenic shock” OR “vasodilatory shock” OR pauci-inflammat*) AND (TITLE-ABS(adult* OR senior* OR aged OR elder* OR man OR woman OR men OR women) OR (LIMIT-TO (EXACTKEYWORD,"Adult") OR LIMIT-TO (EXACTKEYWORD,"Aged") OR LIMIT-TO (EXACTKEYWORD,"Very Elderly") OR LIMIT-TO (EXACTKEYWORD,"Aged, 80 And Over"))) AND NOT INDEX(medline)	129<br><br>-122 duplicates<br><br>=7 unique items	88<br><br>-68 duplicates<br><br>=20 unique items

©2021 Patel P et al. *JAMA Network Open*.

**WHO Global COVID Literature Database**	("multisystem inflammatory syndrome" AND adult*) OR ("multi-system inflammatory syndrome" AND adult*) OR MIS-A OR (Kawasaki* AND Adult*)<br><br>entry_date:([20201214 TO 20210525])	92<br><br>-68 duplicates<br><br>=14 unique items	163<br><br>-124 duplicates<br><br>=39 unique items

Notes: Duplicates were identified using the Endnote automated "find duplicates" function with preference set to match on title, author and year, and removed from your Endnote library. There will likely be additional duplicates found that Endnote was unable to detect.

©2021 Patel P et al. *JAMA Network Open*.

eTable 2. Quality Rating Scheme for Studies (Modified From the Oxford Centre for Evidence-Based Medicine for Ratings of Individual Studies)

Quality Rating Scheme for Studies and Other Evidence	
1	Properly powered and conducted randomized clinical trial; systematic review with meta-analysis
2	Well-designed controlled trial without randomization; prospective comparative cohort trial
3	Case-control studies; retrospective cohort study
4	Case series with or without intervention; cross-sectional study
5	Opinion of respected authorities; case reports

©2021 Patel P et al. *JAMA Network Open*.
